# A Restoration Suitability Index Model for the Eastern Oyster (*Crassostrea virginica*) in the Mission-Aransas Estuary, TX, USA

**DOI:** 10.1371/journal.pone.0040839

**Published:** 2012-07-11

**Authors:** Jennifer Beseres Pollack, Andrew Cleveland, Terence A. Palmer, Anthony S. Reisinger, Paul A. Montagna

**Affiliations:** 1 Department of Life Sciences, Texas A&M University-Corpus Christi, Corpus Christi, Texas, United States of America; 2 Harte Research Institute for Gulf of Mexico Studies, Texas A&M University-Corpus Christi, Corpus Christi, Texas, United States of America; 3 Department of Physical and Environmental Sciences, Texas A&M University-Corpus Christi, Corpus Christi, Texas, United States of America; National Institute of Water&Atmospheric Research, New Zealand

## Abstract

Oyster reefs are one of the most threatened marine habitats on earth, with habitat loss resulting from water quality degradation, coastal development, destructive fishing practices, overfishing, and storm impacts. For successful and sustainable oyster reef restoration efforts, it is necessary to choose sites that support long-term growth and survival of oysters. Selection of suitable sites is critically important as it can greatly influence mortality factors and may largely determine the ultimate success of the restoration project. The application of Geographic Information Systems (GIS) provides an effective methodology for identifying suitable sites for oyster reef restoration and removes much of the uncertainty involved in the sometimes trial and error selection process. This approach also provides an objective and quantitative tool for planning future oyster reef restoration efforts. The aim of this study was to develop a restoration suitability index model and reef quality index model to characterize locations based on their potential for successful reef restoration within the Mission-Aransas Estuary, Texas, USA. The restoration suitability index model focuses on salinity, temperature, turbidity, dissolved oxygen, and depth, while the reef quality index model focuses on abundance of live oysters, dead shell, and spat. Size-specific *Perkinsus marinus* infection levels were mapped to illustrate general disease trends. This application was effective in identifying suitable sites for oyster reef restoration, is flexible in its use, and provides a mechanism for considering alternative approaches. The end product is a practical decision-support tool that can be used by coastal resource managers to improve oyster restoration efforts. As oyster reef restoration activities continue at small and large-scales, site selection criteria are critical for assisting stakeholders and managers and for maximizing long-term sustainability of oyster resources.

## Introduction

Oyster reefs are one of the most threatened marine habitats on earth, with an estimated 15% remaining worldwide [1]. Within the Gulf of Mexico, an estimated 50 to 80 percent of native oyster populations have been lost relative to historic levels [2]. Declines in the abundance of oysters are a consequence of habitat loss due to historical shell dredging [3], water quality degradation [4], disease [5], oil spill effects [6], and hurricanes [7]. In Galveston Bay, Texas, approximately 50%, or 32 km^2^ (8,000 acres) of oysters were lost as a result of Hurricane Ike in 2008 [8]. The resulting sediment deposition smothered live oysters, submerged available hard substrate, and inhibited larval oyster settlement and natural recovery processes. In Louisiana, an estimated 50% of oysters were lost after the Deepwater Horizon oil spill in response to freshwater releases that decreased salinity below oyster tolerance levels [9]. Despite recent and historical losses, there is hope that restoration efforts and adaptive management approaches can revitalize oyster populations in the Gulf of Mexico [10].

**Figure 1:**
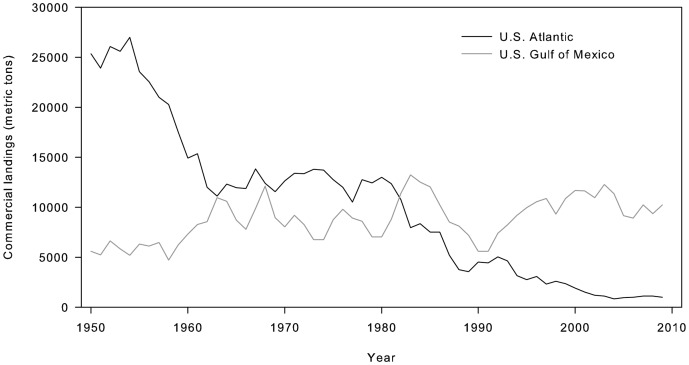
Commercial oyster landings. Commercial oyster landings (metric tons) from the U.S. Atlantic and Gulf of Mexico from 1950–2009 (NOAA 2011).

For successful and sustainable oyster reef restoration efforts, it is necessary to choose sites that support long-term growth and survival of oysters [11], [12]. Selection of suitable sites is an important first step in the restoration process as it can greatly influence mortality factors and may largely determine the ultimate success of the restoration project. Habitat suitability indices are a common tool used by natural resource managers for habitat mapping, conservation and restoration planning [13], [14], [15]. The Gulf of Mexico coast is ideally suited to developing a standardized site selection framework because areas of relatively abundant oyster populations still exist (Fig. 1). In addition, a substantial long-term database is available from the Texas Parks and Wildlife Department's (TPWD) Resource Monitoring Program that describes oyster characteristics and hydrological parameters.

The aim of this study was to develop a restoration suitability index model and reef quality index model to characterize locations based on their potential for successful reef restoration within the Mission-Aransas Estuary, Texas, USA, using a Geographic Information System (GIS)-based approach. GIS is an effective tool that can be used in identifying suitable sites for oyster reef restoration and removes much of the uncertainty involved in the somewhat trial and error selection process that currently exists. The rationale for this study is similar to that used in previous oyster habitat/restoration suitability studies [16], [17], [18], where areas are selected based on the highest potential for successful recruitment, growth, and persistence of oyster populations. Long-term data on oyster populations and environmental variables were integrated within a GIS to characterize locations based on their potential for successful restoration programs.

## Methods

### Study site

**Figure 2:**
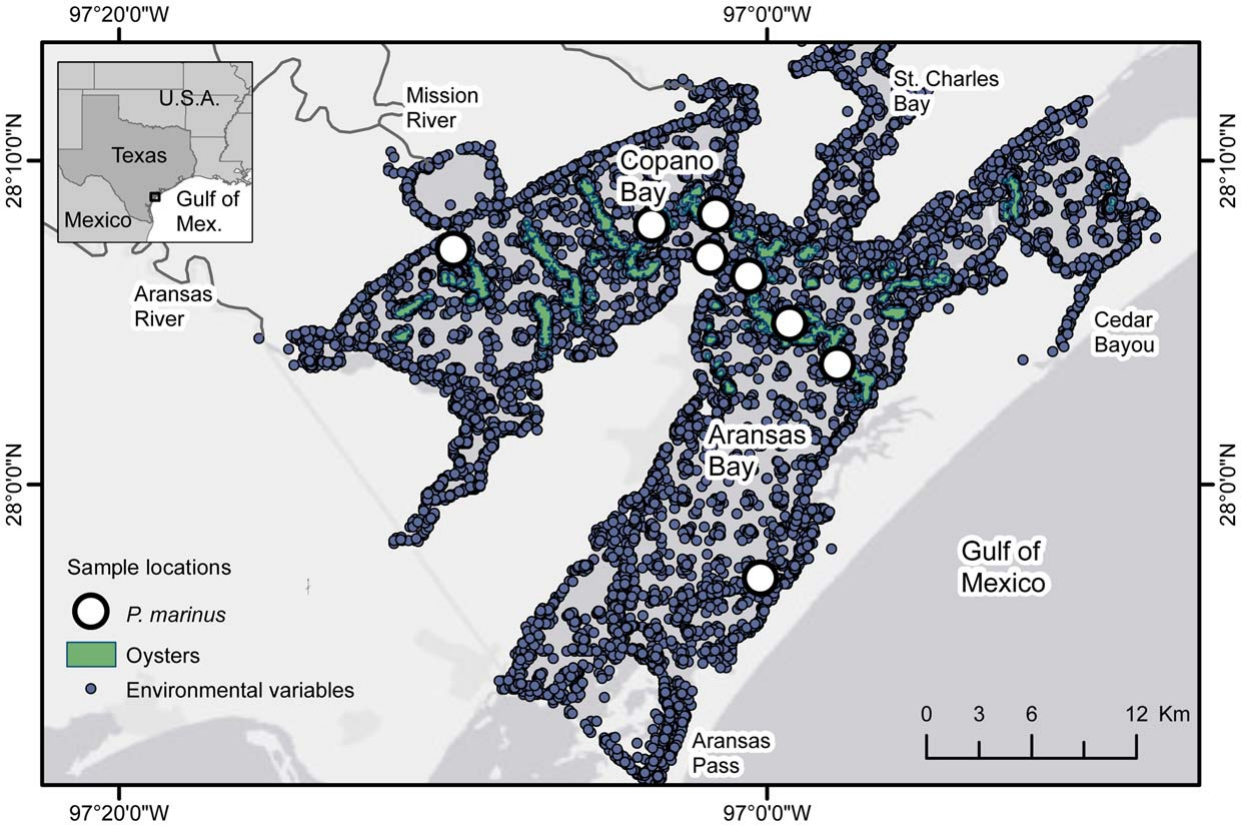
Field sampling locations in the Mission-Aransas Estuary, TX, USA. Sampling locations for oysters (green), environmental variables (gray-blue), and *P. marinus* (white). Environmental variables were also measured at *P. marinus* and oyster sampling locations.

The Mission-Aransas Estuary is a shallow, bar built estuary located in the coastal bend region of the Texas Gulf coast (Fig. 2). The estuary is approximately 540 km^2^ with an average depth of 2 m [19]. The two largest bays in the system are Aransas Bay, which is located closest to the Gulf inlets of Aransas Pass and the intermittently open Cedar Bayou, and Copano Bay, which is located closest to the Mission and Aransas Rivers. The estuary experiences a typical salinity gradient from the river mouths to the Gulf of Mexico, which is driven by episodic freshwater pulses [20]. Oysters occur primarily on large subtidal reefs in the low- to moderate-salinity regions of the estuary, with vertical relief ranging from ∼0.3 to 1.8 m.

### Field surveys

Oysters were collected from January 1986 through December 2009 as part of a fisheries-independent survey conducted by the TPWD Resource Monitoring Program within the Mission-Aransas Estuary (Fig. 2). All necessary collecting permits were obtained from TPWD. Samples were collected by oyster dredge (0.5 m wide, 5 cm diameter mesh) at 20 randomly selected locations on known reefs in Copano Bay and Aransas Bay each month. Latitude and longitude coordinates were recorded using a Garmin GPSMap Geographic Positioning System (GPS) unit. Dredges were towed for 30 s in duration at a speed of 1.3 m s^−1^ for approximately 40 m in distance. Because oysters were collected by oyster dredge, which is a relatively inefficient gear, these data represent a relative index of abundance and oyster size [21]. At each location, 19 live oysters (approx. 95% of all oysters collected) were randomly selected and measured for shell length. A subset of 5 live oysters was also examined for quantification of spat (shell length ≤25 mm) settlement. The amount of dead shell (>25 mm) in each sample was enumerated and a subset of 5 shells was also examined for spat settlement.

Spatially resolved environmental measurements of salinity, dissolved oxygen (mg/l), temperature (°C), and turbidity (NTU) were collected throughout the bay system from January 1975 through April 2009 as part of the TPWD Resource Monitoring Program (Fig. 2). The Aransas Bay bathymetric digital elevation model from the National Oceanic and Atmospheric Administration – National Ocean Service [22] was used to represent depth.

Oysters were sampled quarterly from December 2004 through 2009 and examined for the presence of *Perkinsus marinus*, a protozoan parasite that causes severe mortalities in Gulf of Mexico oyster populations [23]. Ten submarket (26–75 mm) and 10 market-size (≥76 mm) oysters were collected from 8 fixed sampling locations on reefs in Copano Bay and Aransas Bay in each quarterly sampling event (Fig. 1). The southernmost site was discontinued because of difficulties locating live oysters; therefore only 7 sites are reported here. A section of mantle tissue was removed and incubated in thioglycollate medium for 2 weeks following the culture method of Ray [23]. Tissue cultures were stained with Lugol's solution and examined under the microscope. The percentage of oysters infected by *P. marinus* was calculated by dividing the number of oysters infected by the number of oysters tested. Infection intensity was ranked using a 5-point scale (after [24], modified by [25]) from uninfected (0) to heavily infected (5). Weighted prevalence was calculated by ranking the infections on the Mackin Scale and then calculating the average.

### Spatial analysis

Environmental measurements collected by TPWD were imported as point data into a Geographic Information System (GIS; ArcGIS 10, ESRI) and temporally aggregated based on TPWD sampling stations. A new point dataset was created from these aggregations to represent the temporal variability and average conditions at each sampling station. At each TPWD sampling station, mean and standard deviation were generated for salinity, turbidity, and temperature, as well as frequency of occurrence (%) of low dissolved oxygen measurements (<4 mg/l). Dissolved oxygen concentrations of <4 mg/l (instead of 2 mg/l) were selected because all samples were collected during the day and may therefore have resulted in hypoxic conditions at night. New aggregated environmental measurements were spaced 1 minute of longitude on average from each other (approximately 1.8 km), representing the spatial resolution of the TPWD environmental sampling scheme. Although higher resolution data would potentially reveal more localized patterns and processes, finer scale data were not available. However, the available data accurately represent field observations.

Environmental data were spatially interpolated over the area of the estuary. Root mean square error was used to compare and select interpolation methods. The best interpolation method was local polynomial interpolation (1^st^ order polynomial with barriers) weighted by temporal frequency of sampling each station. This model is a good candidate for mapping data regularly collected from the environmental monitoring networks [26]. Rasters were created using a 0.1 minute cell size (approximately 180 m) for mean and standard deviation of salinity, temperature (°C), and turbidity (NTU), as well as frequency of occurrence (%) of low dissolved oxygen measurements (>4 mg/l). For depth, the Aransas Bay bathymetric digital elevation model from NOAA-NOS was resampled to 0.1 minute cell size to match the spatial resolution and extent of the environmental variable rasters. Sediment grain size data available [27] had low spatial resolution and sampling points were avoided on reef areas, and therefore were not useful in the current study. Side scan sonar and sub-bottom profiling data were not available for the entire study area so were also not used.

Oyster data (except for disease data) from TPWD were imported as point data into the GIS. Where oyster samples were collected with the presence of live oysters, points were buffered by 80 m (the area covered by dredge sampling) to create polygons that represent live oyster reef. Live reef polygons were then used to aggregate oyster sampling data, where mean abundance of live oysters (>25 mm shell length), dead shell (>25 mm shell length), and spat (5–25 mm shell length) on live oysters and dead shell were calculated for each continuous reef polygon.

**Figure 3:**
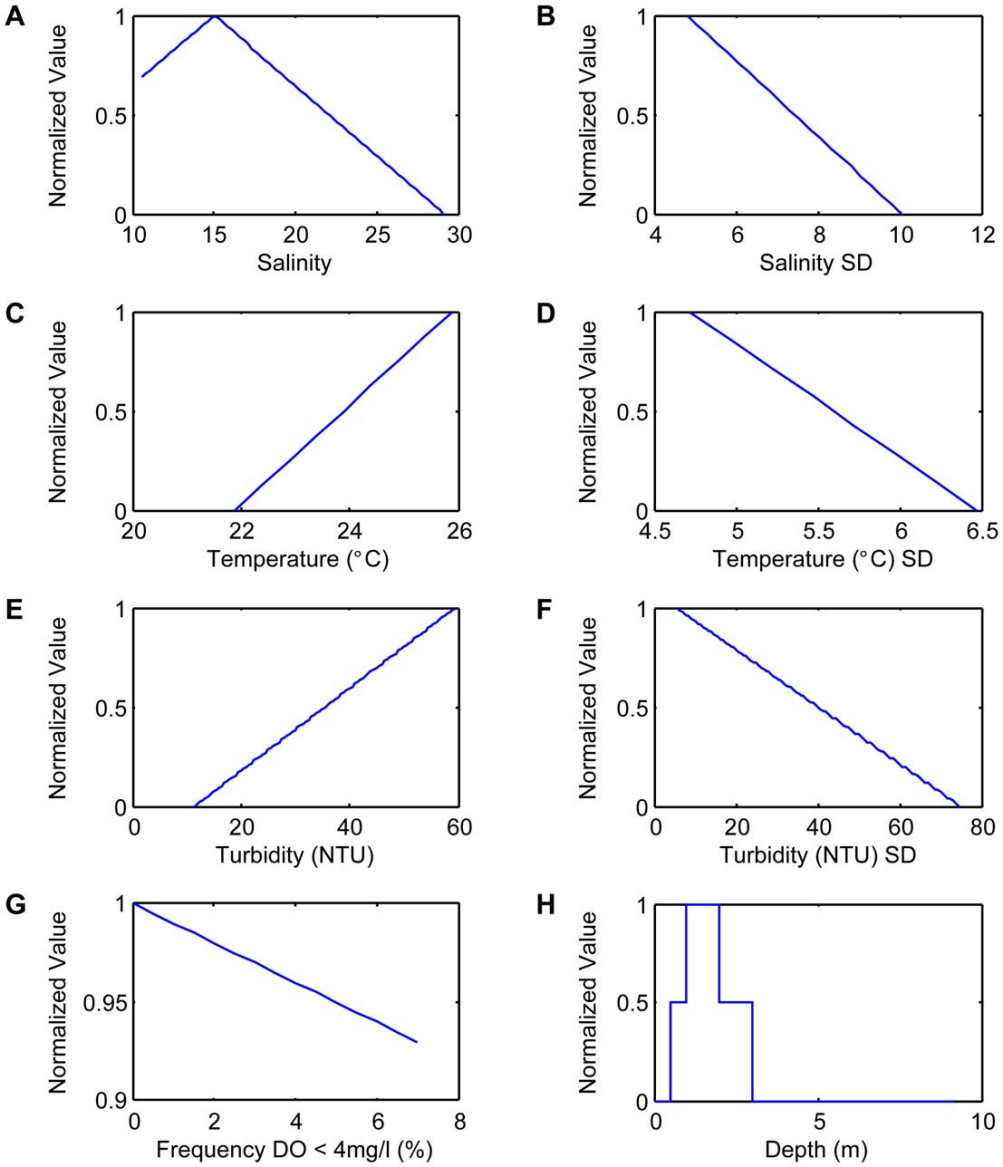
Suitability index graphs for model variables. Relationship between actual values of environmental variables and their corresponding restoration suitability values for *Crassostrea virginica;* values of 1 are optimal, and values of 0 are unacceptable.

As for the environmental variable rasters, data were normalized by scaling from 0–1, where values of 1 are optimal, and values of 0 are unacceptable [17], [18], [28]. Specific environmental values used for generating the restoration suitability index are presented in Fig. 3.

Mean turbidity data were normalized using the following equation:
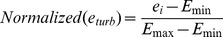
(1)where *E_min_* and *E_max_*  =  the minimum and maximum values, respectively, for the variable E.

Higher values were assigned to high turbidities to maximize available suspended food particles.

Mean temperature data and standard deviation of temperature, salinity, and turbidity were normalized using the following equation:
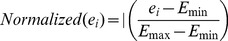
(2)where higher values were assigned to lower temperatures because the prevalence and intensity of *P. marinus* infections increase with increasing temperature [29]. Higher values were assigned to low standard deviations to optimize areas with less environmental variability.

Mean salinity data were normalized using the following equation:
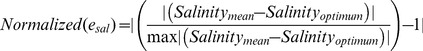
(3)where the optimum salinity value was 15. At salinities ranging from 10–20, oysters are present in dense populations, have high reproductive ability, and are subject to relatively lower densities of predators and disease [30]. Higher values were assigned to moderate salinities because the relationship with oysters is nonlinear; moderate salinities are more favorable than are low or high levels.

Frequency of DO <4 mg/l was normalized using the following equation:
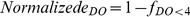
(4)where *ƒ_DO<4_* is the frequency of dissolved oxygen <4 mg/l. Higher values were assigned to lower frequencies.

Continuous depth values were given 3 different index values. Depths of 1–2 m were given a value of 1 because of the feasibility of large-scale restoration using barges as well as the success of past restoration efforts within this depth range in the Mission-Aransas Estuary. Depths between 0.5 and 1 m were given a value of 0.5 because of suitability for oyster growth but limitations to construction methods. Depths of 2–3 m were also given a value of 0.5 because of suitability for oyster growth, but also increased construction costs and observations of higher proportions of fine sediment accumulation at these depths within the Mission-Aransas Estuary. Depths of <0.5 and depths of >3 m were given values of 0 because of being too shallow or too deep for long-term reef sustainability as observed in previous unsuccessful restoration efforts in this estuary.

The normalized environmental rasters were then combined using a weighted geometric mean function [17], [18] using the equation:
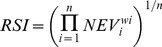
(5)where *w_i_* is the relative weight of importance of the normalized environmental variables (NEV), producing a restoration suitability index (RSI). Using this method, the overall suitability at a specific location is given a ranked value of 0 if any single parameter is unsuitable (has a value of 0). All NEV except salinity were given equal weights of 2 and all standard deviations were given equal weights of 1. Salinity plays the most important role in oyster health in the study area and thus was given a corresponding weight of 4.

The sensitivity of the model to particular environmental variables was assessed using the following equation:
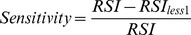
(6)where *RSI* is the index of restoration suitability (Eq. 5) and *RSI_less1_* is RSI with an individual normalized environmental variable (NEV) removed, one at a time. The output of each scenario is presented in map form to allow for visual comparison of percent change in the base model resulting from removal of an individual variable.

The ratio of live oysters to total (live plus dead) was calculated. Mean abundance of spat was scaled from 0 to1 using Eq. 1. An index of oyster reef quality (*Index_rq_*) was then derived using the following equation:
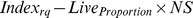
(7)where *Live_Proportion_* is the ratio of live oysters to total and *NS* is the normalized spat value. Higher values of the index indicate oyster reefs with higher quality oyster populations (abundant live oysters and spat, moderate to few dead oysters).

*Perkinsus marinus* sampling occurred at fixed sampling stations throughout the Estuary; therefore these data were not integrated into the model, but were mapped as a series of bar charts to illustrate disease trends along a salinity gradient. *Perkinsus marinus* accumulates in oyster tissue over time and infections tend to be size-specific, with large oysters having higher infection levels and disease-related mortality than small individuals [31]. Therefore, *P. marinus* data were presented using percent infection and weighted prevalence of both submarket (<76 mm) and market (≥76 mm) size classes to demonstrate size-specificity.

## Results

**Figure 4:**
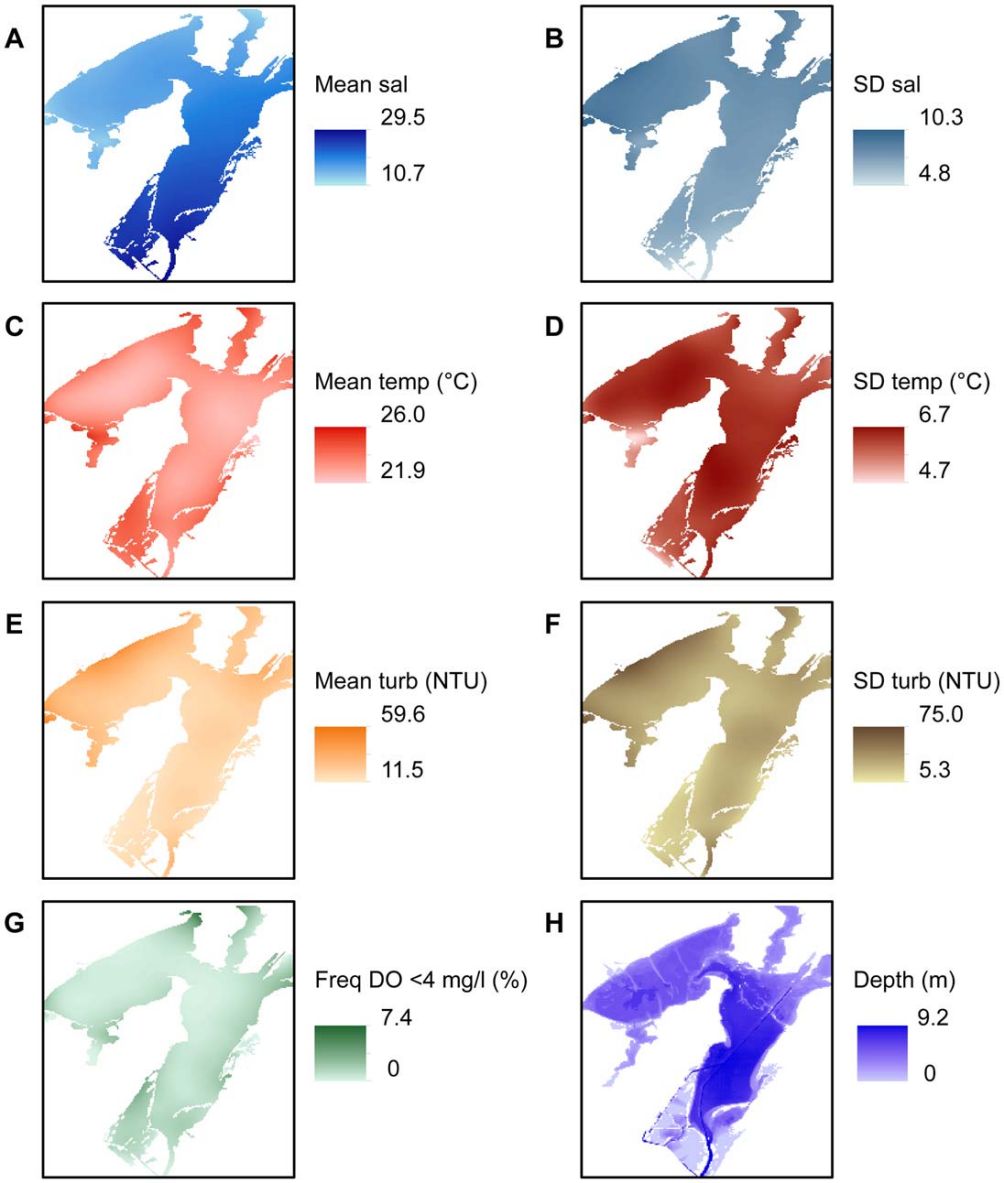
Spatially interpolated environmental measurements in the Mission-Aransas Estuary, TX, USA. Mean (left column) and standard deviation (SD, right column) for salinity (sal; A and B), temperature (temp; C and D), and turbidity (turb; E and F). Frequency of dissolved oxygen <4 mg/l (G); and depth (H).

Environmental conditions were variable over the period of the study. Mean salinities ranged from 10.7–29.5 (Fig. 4A). Lower salinities were observed in the secondary bays (Copano and St. Charles) due to river inputs while higher salinities were observed in Aransas Bay, located closest to the Gulf of Mexico inlets. The highest salinity variability was observed in the northwest quadrant of Copano Bay, which is influenced by sporadic pulses of freshwater from the Mission and Aransas Rivers (Fig. 4B). Mean temperatures ranged from 21.9–26.0°C and were highest in the shallow margins of the estuary, adjacent to the slow-moving Aransas River and Copano Creek, and throughout relatively shallow St. Charles Bay (Fig. 4C). The highest temperature variability was observed in the central portion of Copano Bay, which is subdivided by several long oyster reefs that likely constrain water circulation (Fig. 4D). Mean turbidities ranged from 11.5–59.6 NTU and were highest and more variable along the northwestern edge of Copano Bay, where the Mission and Aransas Rivers and Copano Creek drain into the estuary (Fig. 4E, F). The frequency of low dissolved oxygen (<4 mg/l) measurements was fairly small, ranging from 0–7.4% (Fig. 4G). Low dissolved oxygen levels were more frequent in the shallow portions of the estuary. Mean water depth ranges from 0–9.2 m (Fig. 4H). The deepest water occurs in the Gulf Intracoastal Waterway and in the southern portions of Aransas Bay, and the shallowest water exists in areas of existing oyster reef.

**Figure 5:**
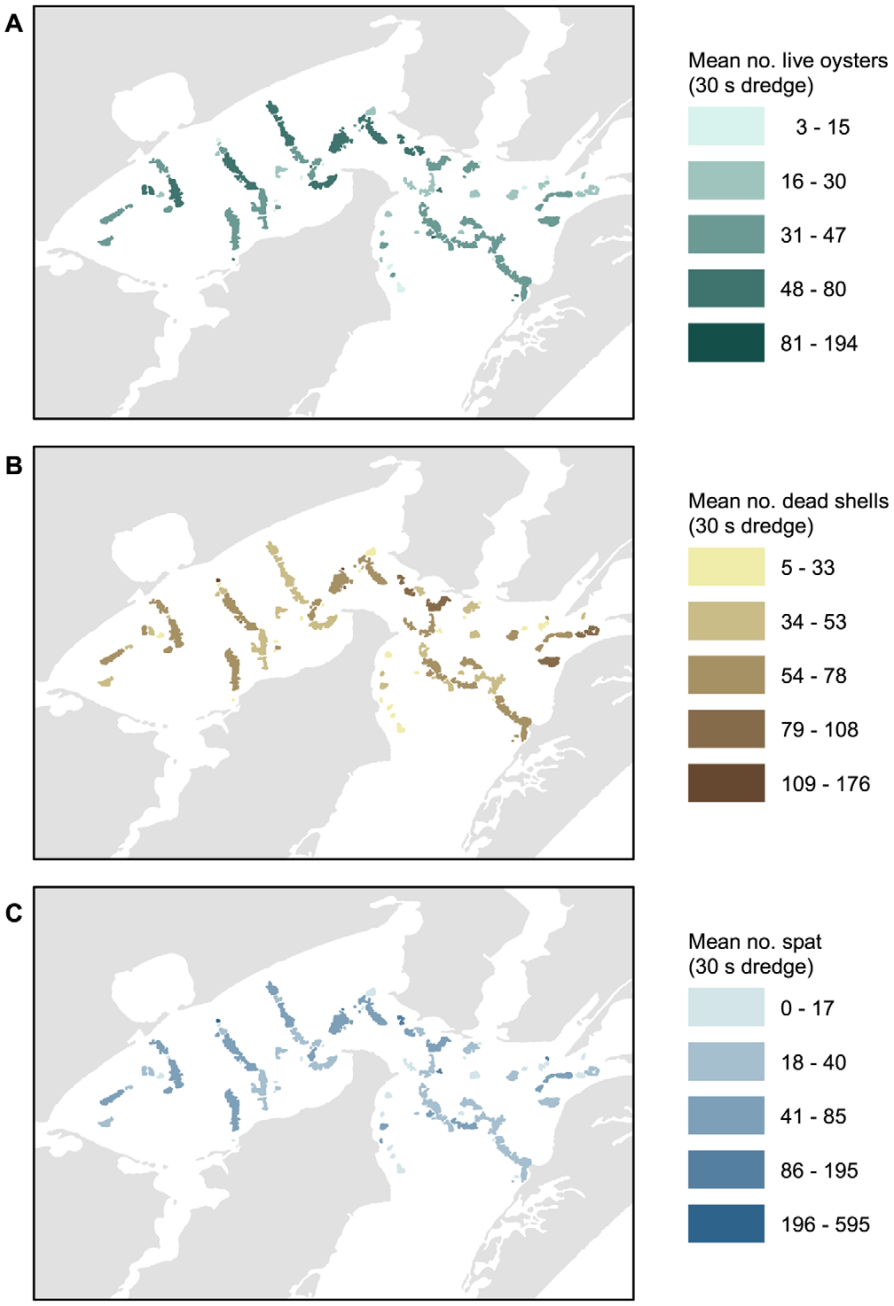
Oyster condition on reefs in the Mission-Aransas Estuary, TX, USA. Mean number of A) live oysters (≥26 mm), B) dead oyster shells ≥26 mm, and C) spat (5–25 mm) collected using 30 second dredge (1986–2009).

Live oyster abundance was highest (>80 oysters per 30 s tow) on several reefs in Copano Bay and at the confluence of Copano and Aransas Bays (Fig. 5A). Live oyster abundance was moderate to low (3–47 oysters per 30 s tow) throughout Aransas Bay. Moderate numbers of dead shell (>25 mm shell length) were observed throughout both Copano and Aransas Bays (Fig. 5B). Spat abundance was highest on several reefs in Copano Bay and moderate to low throughout Aransas Bay, similar to the distribution of live oysters (Fig. 5C).

**Figure 6:**
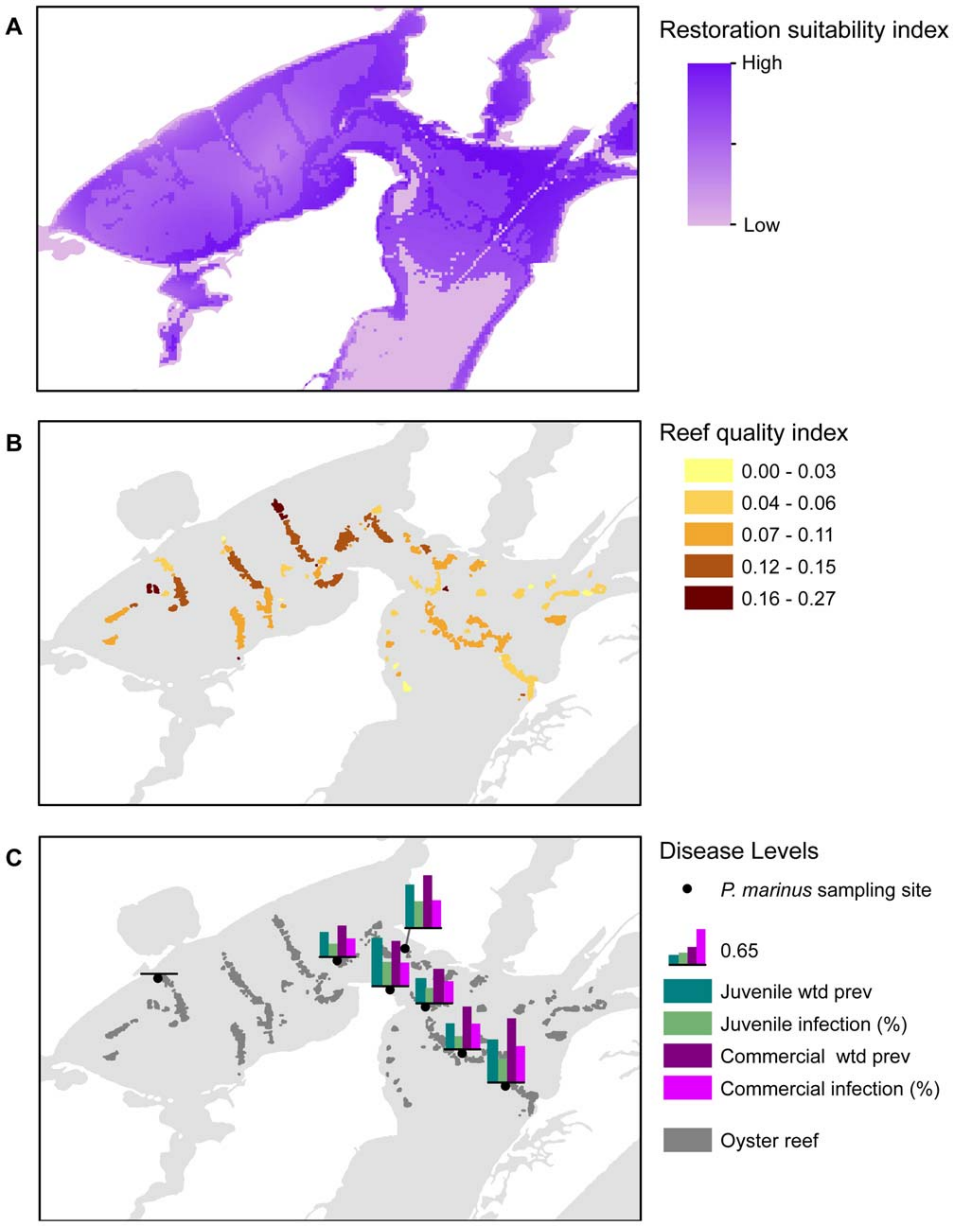
Restoration suitability index and reef quality index. A) Restoration suitability index. Darker shading indicates higher index value. B) Reef quality index. Dark shading indicates higher index value. C) *P. marinus* disease levels: at each of 7 sampling stations, bar graphs illustrates percent infection and weighted prevalence for juvenile (<76 mm shell length; green-hued color bars) and commercial oysters (≤76 mm shell length; pink-hued bars).

The restoration suitability index illustrates the range of suitability of environmental variables throughout the Mission-Aransas Estuary (Fig. 6A). Highest index values often correspond with areas of existing oyster reef. Lowest index values occur in the southern, deeper portions of Aransas Bay and in the Gulf Intracoastal Waterway. The reef quality index quality was highest on reefs in Copano Bay (Fig. 6B). Along the 7 fixed sampling sites, *Perkinsus marinus* infection levels and weighted prevalence were highest for juvenile oysters (<76 mm shell length) at the southern-most location in Aransas Bay and at the confluence with Copano Bay (Fig. 6C). Infection levels and weighted prevalence of *P. marinus* in commercial oysters (≥76 mm shell length) were generally higher than in juvenile oysters. Weighted prevalence for commercial oysters was consistently high (>0.50) over 6 of the 7 sites and low (<0.26) at the upstream-most sampling station. The lowest disease levels for both juvenile and commercial oysters were observed at the upstream-most location.

**Figure 7:**
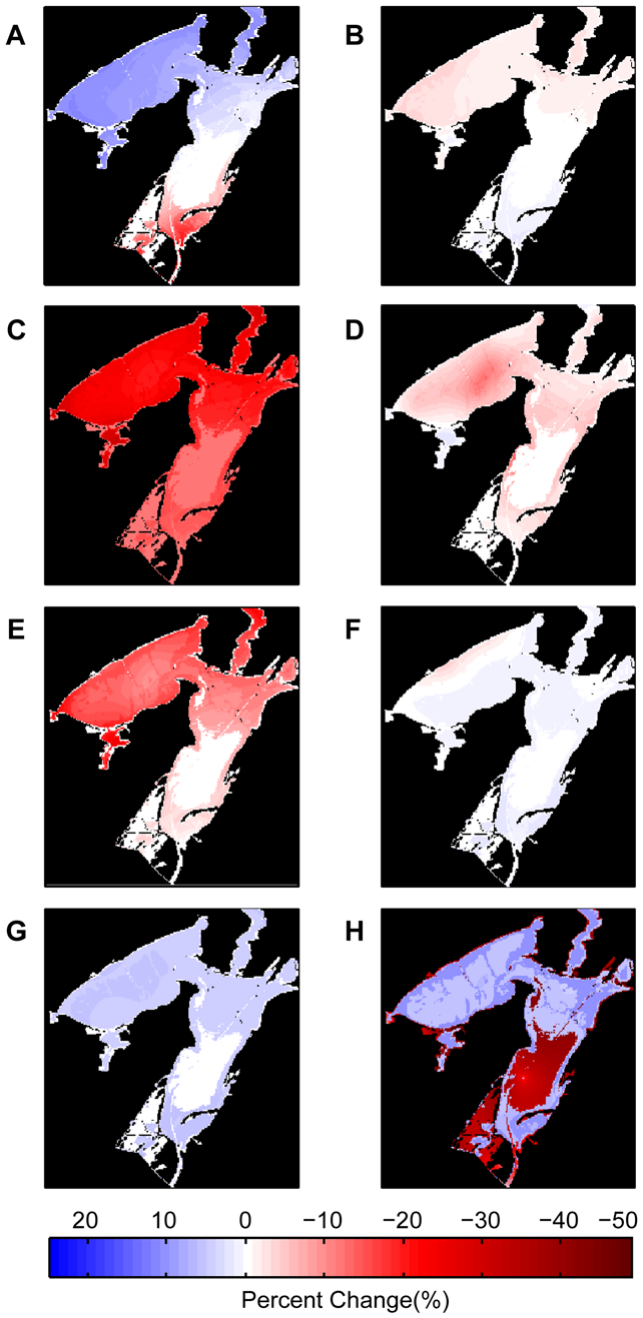
Sensitivity analyses. Percent change from the restoration suitability index base model due to removal of individual normalized environmental variables, one at a time. Removal of: A) mean salinity, B) standard deviation (SD) salinity, C) mean temperature, D) SD temperature, E) mean turbidity, F) SD turbidity, G) frequency of low dissolved oxygen; H) depth.

The sensitivity analysis illustrates the percent change from the base restoration suitability index model when individual normalized environmental variables were removed, one at a time (Fig. 7). Removing mean salinity, mean temperature, or mean turbidity resulted in the greatest percent change in Copano Bay (Figs. 7A, C, E). Removing standard deviation of salinity, temperature, or turbidity, or frequency of low dissolved oxygen, resulted in relatively small changes from the base model throughout the estuary (Figs. 7B, D, F). Removing depth resulted in the greatest percent change in the southern, deeper portions of Aransas Bay.

## Discussion

For successful and sustainable oyster reef restoration efforts, it is necessary to choose sites that support long-term growth and survival of oysters. In general, this requires suitable environmental conditions, adequate larval supply, sustained survival and growth of juvenile and adult oysters, and low disease levels. Proper site selection is one of the most important decisions that restoration groups have to make. Selection of suitable sites is critical as it can greatly influence mortality factors and may largely determine the ultimate success of the restoration project. The application of GIS provides an effective methodology for characterizing locations based on their potential for successful reef restoration and removes much of the uncertainty involved in the sometimes trial and error selection. This GIS-based approach also provides an objective and quantitative tool for planning future oyster reef restoration efforts.

In this study, we sought to characterize locations within the Mission-Aransas Estuary based on their potential for successful oyster reef restoration. The justification for selecting sites based on their potential for successful recruitment, growth, and survival follows that used in previous restoration/habitat suitability studies [16], [17], [18]. Depending on project goals, map layers could be integrated in different ways to answer different questions or apply adaptive management strategies. For example, areas with relatively low spat abundance could be targeted for restoration using spat-on-shell [32], reefs with high levels of disease could be targeted for restoration using disease resistant oysters [33], and areas with persistent hypoxia could be restored using reefs with higher vertical relief [12]. This flexibility may be useful to resource managers in dealing with local-scale issues in otherwise high-quality environmental conditions.

In the Mission-Aransas Estuary, it was strongly desired to select sites with moderate salinities to reduce oyster mortalities associated with the high and low ends of the salinity range. The Estuary is located in a semi-arid climate and regularly experiences prolonged droughts. Severe oyster mortalities can result from low flow periods as predators with higher salinity optima, such as the southern oyster drill *Stramonita haemastoma* and the stone crab *Menippe mercenaria* are favored [34], [35]. In addition, *Perkinsus marinus* disease initiation and progression are favored by high salinities in combination with high temperatures [24], [36]. However, it has also been suggested that it is critically important to protect and restore oyster populations in mesohaline waters, regardless of disease presence, to encourage the development of disease resistant populations [37]. At this time, no such *P. marinus*-resistant populations have been identified within the Mission-Aransas Estuary. At the low end of the salinity range, the Mission-Aransas Estuary is strongly driven by episodic freshwater pulses that depress and then maintain low salinities for a prolonged period [20]. When exposed to sustained periods of low salinities, oysters may reduce their feeding and growth rates, depress or arrest gametogenesis, delay spawning, and/or resorb gonadal material [38], [39], [40], which can lead to recruitment failure or post-settlement mortality. In general, within the Mission-Aransas Estuary, selecting restoration sites with historically moderate salinities (∼15) and low salinity variability will favor oyster growth and survival over predators and disease [41].

For oyster reef restoration in the Mission-Aransas Estuary, it was also desirable to select areas with low to moderate temperatures. Oysters in Gulf Coast estuaries experience extended periods of warm temperatures compared to northern USA estuaries. Because of the positive correlation with *P. marinus* infection, high temperature values were reclassified with low rankings. Reproduction of *P. marinus* increases at temperatures above 20°C and the parasite proliferates rapidly at temperatures of 25°C or higher [42], [43]. Because water temperatures in Gulf of Mexico estuaries generally exceed 20°C for 6 months of the year, oysters do not receive the long reprieve from disease pressures experienced by oysters in northern USA and other high latitude estuaries [30]. Although temperature is a more difficult environmental parameter to control in terms of restoration site selection, particularly in sub-tropical and tropical regions, it is advisable to select locations with sufficient circulation to limit temperature extremes and reduce the probability of disease related mortalities.

Dissolved oxygen concentrations in estuarine waters are another critical component of water quality affecting the survival of oysters. Although the Mission-Aransas estuary has not historically experienced low dissolved oxygen concentrations (Fig. 4G), many estuaries (e.g. Chesapeake Bay) exhibit seasonal salinity and temperature stratification that can lead to hypoxic or anoxic bottom waters. Early developmental stages of oysters may experience negative effects on survival and feeding due to prolonged reductions in dissolved oxygen concentrations [44], whereas adult oysters are better able to survive extended anoxia through the use of anaerobic metabolic pathways [45]. However, the potential negative effects of bottom water hypoxia may be overcome through reef design, particularly increasing reef height, which can provide refuge for oysters and other reef organisms above hypoxic bottom waters [46]. Nevertheless, it is advisable to consider historical frequency of low dissolved oxygen concentrations/hypoxia when selecting the location for (and design of) a restoration site [47].

The Mission-Aransas Estuary experiences a wide range of turbidities; higher values are generally associated with windy conditions in the spring (personal observation). Although previous laboratory studies have reported reduced oyster feeding activities under high turbidity conditions [48], these studies examined much higher levels of suspended solids than present in this system. Conversely, oysters in the Mission-Aransas Estuary appear to experience increased stress and during low turbidity periods, perhaps as a response to low food availability (personal observation; SM Ray, personal communication). Because only 15–30% of surface inflows to the system are from rivers [20], [49], the primary driver of turbidity is via re-suspension of bottom sediments. The high amount of re-suspension is due to a combination of seasonal winds and a shallow water column.

The GIS approach employed in this study to characterize locations based on their potential for successful reef restoration in the Mission-Aransas Estuary is flexible and provides a mechanism for considering alternative approaches. Coastal resource or fishery managers can compare different preferred ranges of values for each variable, develop alternative restoration strategies based on these choices, and visualize the effects of these alternative strategies in real time.

The current study has attempted to include as much available and relevant information as possible in the analysis of sites for oyster reef restoration in the Mission-Aransas Estuary. Nevertheless, the approach could be refined further with additional information. For example, side scan sonar and sub-bottom profiling methods are being used more frequently to characterize the bottom and sub-bottom conditions of estuarine systems [50], [51], [52]. These data have been collected for a portion, but not all, of the Mission-Aransas Estuary and thus were not able to be included in this study [53]. Side scan sonar can cover large areas quickly and can produce a detailed picture of the bay bottom, which is useful for identifying the distribution and scale of oyster reefs and other surficial features. Sub-bottom profilers provide additional information about the shallow structure of the bay bottom, which is important for identifying the presence of hard substrates for supporting reef building materials. New reef materials may sink or experience high rates of sedimentation if an appropriate location is not selected [54]. In estuaries where bay bottom conditions are unknown, it is advisable that these methods be considered and utilized in advance of site selection.

In general, the restoration suitability index model matches the current distribution of oyster reef throughout the Mission-Aransas Estuary. However, there are some differences, particularly in the NE region of the system, where the restoration suitability index predicts favorable conditions for oysters, yet there are few reefs. The reason behind this mismatch may be due, in part, to missing information on bay bottom conditions. Particularly within Aransas Bay, these areas tend to have softer sediments (personal observation) and therefore may not facilitate oyster reef development. Future collection of comprehensive side scan sonar and sub bottom profiling data throughout the Mission-Aransas Estuary and inclusion in the model could improve restoration suitability index results. In addition, we have ongoing restoration efforts throughout the estuary, and thus will be able to empirically test the suitability of the selected regions.

This study sought to support oyster reef restoration efforts by developing a GIS-based methodology for *a priori* improvement of restoration success via an informed site selection process. This approach provides an interactive and quantitative tool for planning future oyster reef restoration efforts. The proposed restoration suitability model could be further refined when additional data (e.g. sub-bottom conditions) become available. Although it is critically important to continue restoring degraded habitats, it is also essential to develop standardized, science-based tools to inform this process. This model provides a practical, objective, and quantitative decision support tool for assisting stakeholders and managers in planning for future oyster reef restoration efforts and for maximizing long-term sustainability of oyster resources.
